# Automation of Monte Carlo‐based treatment plan verification for proton therapy

**DOI:** 10.1002/acm2.12923

**Published:** 2020-05-26

**Authors:** Maduka Kaluarachchi, Vadim Moskvin, Fakhriddin Pirlepesov, Lydia J. Wilson, Fang Xie, Austin M. Faught

**Affiliations:** ^1^ Department of Radiation Oncology St. Jude Children's Research Hospital Memphis TN USA

**Keywords:** automation, Monte Carlo, proton therapy

## Abstract

**Purpose:**

Independent calculations of proton therapy plans are an important quality control procedure in treatment planning. When using custom Monte Carlo (MC) models of the beamline, deploying the calculations can be laborious, time consuming, and require in‐depth knowledge of the computational environment. We developed an automated framework to remove these barriers and integrate our MC model into the clinical workflow.

**Materials and Methods:**

The Eclipse Scripting Application Programming Interface was used to initiate the automation process. A series of MATLAB scripts were then used for preprocessing of input data and postprocessing of results. Additional scripts were used to monitor the calculation process and appropriately deploy calculations to an institutional high‐performance computing facility. The automated framework and beamline models were validated against 160 patient specific QA measurements from an ionization chamber array and using a ±3%/3 mm gamma criteria.

**Results:**

The automation reduced the human‐hours required to initiate and run a calculation to 1–2 min without leaving the treatment planning system environment. Validation comparisons had an average passing rate of 99.4% and were performed at depths ranging from 1 to 15 cm.

**Conclusion:**

An automated framework for running MC calculations was developed which enables the calculation of dose and linear energy transfer within a clinically relevant workflow and timeline. The models and framework were validated against patient specific QA measurements and exhibited excellent agreement. Before this implementation, execution was prohibitively complex for an untrained individual and its use restricted to a research environment.

## 1 INTRODUCTION

Proton therapy is becoming an increasingly common treatment modality in radiation oncology.^1^ As this technology matures, consensus guidelines continue to be developed for many proton therapy centers. Although currently there are clear guidelines on the use of independent dose calculations to verify the dose in 3D and intensity‐modulated treatment photon therapy,^2^ at the time of writing this manuscript, such guidelines did not exist for proton therapy. Due to lack of independent commercial solutions, proton centers often develop their own in‐house Monte Carlo (MC)^3^, ^4^, ^5^ or analytical^6^ second check systems.

Although most commercial treatment planning systems (TPS) have started releasing MC‐based dose calculations, analytical pencil beam algorithms (PBA) are still widely used. Analytical algorithms are highly accurate in predicting the dose in homogeneous regions, but can fail in regions of heterogeneities.^7^ When heterogeneities exist in the path of the proton beam, the Bragg peak is degraded and it affects the proton fluence and width of the distal fall‐off region of the Bragg peak.^8^ Goitein and Sisterson,^9^ Urie et al.,^10^ and Sawakuchi et al.^11^ reported that the main cause for degradation of the Bragg peak is multiple Coulomb scattering, which is not taken into account in PBA dose estimations. A recent publication from the Imaging and Radiation Oncology Core Quality Assurance Center, Houston (IROC‐H),^7^ demonstrated the inaccuracy of PBA in predicting the dose in anthropomorphic lung phantoms used in its remote auditing program. Differences observed between PBA and measured dose distributions were due to density heterogeneities close to the bone‐air and tissue‐air interfaces.

Unlike PBAs, MC calculations include explicit modeling of particle transport, which increases the accuracy of dose calculations.^4^, ^12^ Explicit transport of particles allows MC simulations to perform more accurate dose calculations in regions of heterogeneities. The IROC‐H study^7^ compared dose calculations in the lung phantom among MC, PBA, and film measurements. Dose differences between PBA and film measurements were as high as 30%, whereas the maximum dose difference between MC and film measurements was 12%. Accuracy of the MC technique, coupled with the observed differences between clinical dose calculations, suggest that an MC‐based second check system is well‐suited to identify cases in which limitations of the TPS algorithms are clinically relevant.

Due to concerns of linear energy transfer (LET) correlating with treatment‐related complications,^13^, ^14^ a prospective evaluation of LET distributions before treatment would be useful in establishing best practices with regard to beam arrangement, spot placement, and spot weighting.^15^ LET can be easily scored in MC calculations and integrated into the independent secondary check.^5^

MC‐based patient‐specific dose calculations require the execution of several steps before and after MC simulations. When the process is executed manually and in a stepwise manner, it can be time consuming and prone to human error. Increased computational power and improved access, and even direct integration to TPS data with scripting, have enabled workflow automation in the treatment planning process,^16^ including the use of secondary dose checks.^17^, ^18^, ^19^

In this study, we develop a framework for an automated MC dose/LET verification system that reduces the person‐hours required to perform an MC calculation. We present a workflow that can be implemented at any proton therapy center that uses spot scanning. Furthermore, we focus on studying dose and LET distributions in pediatric patients. If properly developed, an MC‐based system can provide an excellent independent secondary check of the calculated dose and also calculate LET distributions before treatment with minimal workflow disruption.

## 2 MATERIALS AND METHODS

A framework was developed to fully automate the MC dose calculation process for patient treatment plans. All calculations were initiated by the Eclipse Scripting Application Programming Interface (API) in the Eclipse Treatment Planning System (Varian Medical Systems, Palo Alto, CA, USA). The API passed jobs to a staging server, from which jobs were submitted to a set of clustered computational nodes running Red Hat Enterprise Linux v6.5 (Red Hat) facilitated by an institutional high‐performance computing (HPC) facility. The staging server is a dedicated virtual machine hosted by VMware vSphere 6.5 (VMware, Palo Alto, CA, USA) and was built with 2 Intel Xeon CPU 35‐2687W v4 processors (Intel, Santa Clara, CA, USA) and Windows Server 2016 Standard Edition (Microsoft Corp., Redmond, WA, USA). Scripts to facilitate data transfer and exchange on the server were written using Perl v5.24.1. A set of functions written in MATLAB (Mathworks, Natick, MA, USA) were used to execute each stage of pre‐ and postprocessing of MC dose calculations (sections 2.B and 2.D respectively). For comparisons with TPS calculations, the Eclipse Pencil Convolution Superposition v13.7.15 algorithm was used.

**Fig. 1:**
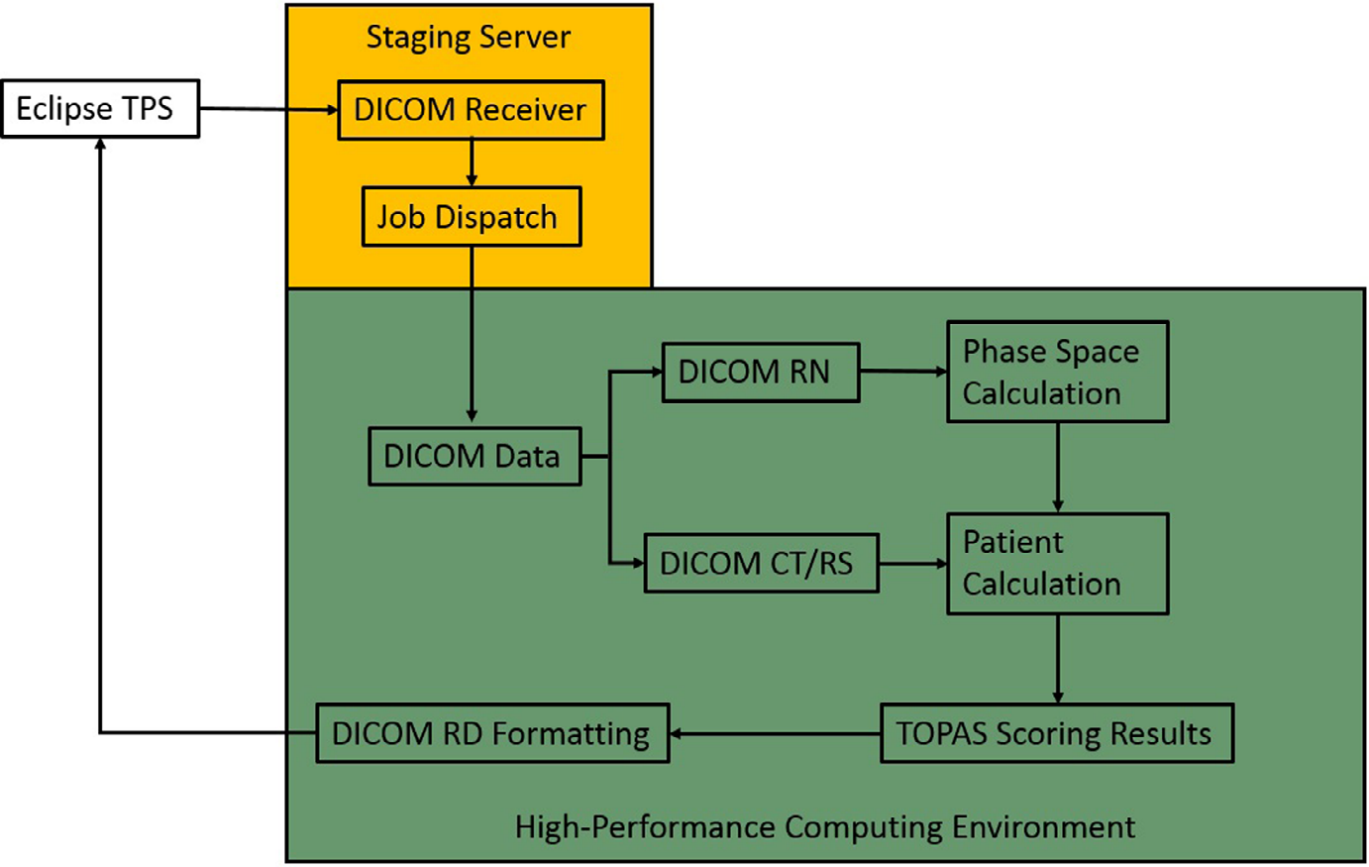
Automation Workflow – User interaction is limited to the scripting application programming interface in the treatment planning systems. All actions within the staging server and high‐performance computing environment are automated. DICOM CT and RS are the imaging and structure files, respectively, while DICOM RN is the plan file

The following sections provide details of each step in the automation process. Figure 1 summarizes the automated steps and the environment in which the steps occur.

### 2.A DICOM import and export

The automation process starts when the user exports DICOM files associated with a patient plan from the clinical TPS to the server. By passing DICOM data through a server, the identity of the user (sender) is recorded and used to notify the user of results of the data integrity check via e‐mail. During the data integrity check, patient data are verified and any missing data required for the MC simulation are identified. Scripts on the server automatically detect the result of the integrity check and launch the next stage of the process. After the simulation is complete, output data and their relevant metadata are sent back to the server and stored in a database that can later be accessed through a web interface or imported to the dose visualization software of choice (e.g., TPS).

### 2.B Preprocess

To perform calculations on patient anatomy, the MC code generates a voxelized three‐dimentional (3D) patient geometry on the basis of computed tomography (CT) images of the patient. As part of the automation process, CT images undergo a preprocessing stage, in which the original CT images are modified to better reflect the conditions during treatment. Modification is done in several steps. First, a model of the treatment couch is burned into the CT image, thereby replacing the simulation couch. Because of different physical geometries and water equivalent thicknesses, using the treatment couch model is important for patients receiving posterior beams.

Voxels outside the patient's body contour are overridden to a Hounsfield unit value of –1000, which corresponds to air. The override to air removes external artifacts, warm blankets, or other items that are either not present during treatment or may affect the accuracy of calculations. Additional overrides corresponding to surgical clips or range‐shifting devices are burned into the image to reflect overrides chosen in the clinical TPS.

**Fig. 2:**
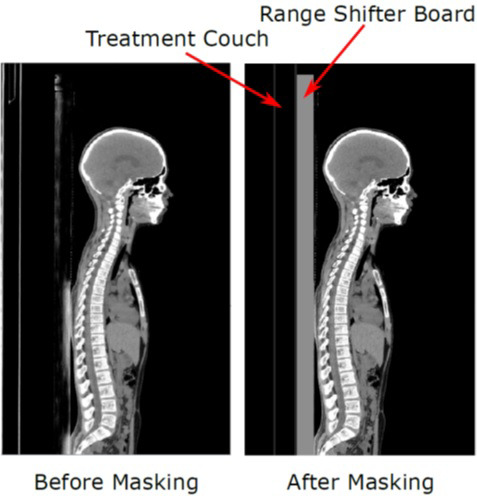
Preprocessing of CT Image – Sagittal view of a CT before (left) and after (right) preprocessing. In the preprocessed image, the simulation couch is replaced by the treatment couch and the range shifter board used for treatment is added to the image

Figure 2 shows an example of a CT image before and after the preprocessing step. The CT image on the left shows the original image, and that on the right shows the image after removing the simulation couch and adding the treatment couch and range shifter board. The preprocessed CT image is used to calculate the dose in MC simulations.

### 2.C Monte‐Carlo simulation

The TOol for PAarticle Simulations (TOPAS)^20^ is an MC tool specially built for proton therapy simulations based on the general purpose MC code GEANT4.^21^ TOPAS has been used for all simulations in the automated MC system. The previously developed two‐stage MC model for simulating treatment with the PROBEAT‐V spot scanning proton therapy system was used as the computational engine for the automated system.^22^ In the first step, the Hitachi PROBEAT‐V beamline and nozzle were simulated, and phase‐space files were generated with TOPAS v2.^22^ DICOM RT plan data were used to create a set of input files for TOPAS simulations, with the MATLAB script written in‐house. To simulate the beamline the TOPAS input contains the scanning magnet parameters, beamline energy parameters, and spot fluence parameters upstream of the scanning magnets.

Each proton field was simulated by using 2 × 10^8^ proton histories. For the given number of histories, the statistical dose uncertainty at the center of a 10 cm spread out Bragg peak was estimated to be 0.6% at one standard deviation. The total number of proton histories was split among 50 parallel jobs to save time. To change the event sequence the initial seed was altered for each of the jobs. At the end of the beam nozzle, a phase‐space scorer was used to obtain the position, particle type, energy, and momentum of particles crossing that surface and was saved in binary format.

In the second step, the generated phase‐space particles were used in the calculation on the CT‐based patient geometry located downstream of the nozzle. TOPAS v3.1.3 was used to simulate particle transport by using patient geometry. Information such as the location of CT images, isocenter, and CT machine–dependent density correction factors were also included in the TOPAS input files. To score physical dose and dose‐averaged LET (LET_d_) in the CT‐based patient geometry, usually split into 512 × 512 × (number of CT slices) voxels, “DoseToMedium” and “ProtonLET” tallies were used respectively. At the end of the simulation, each parallel job generated corresponding dose and LET_d_ files in binary format.

### 2.D Postprocess

The system detected the end of the simulation stage by tracking the job ID issued by the HPC facility when the job was originally submitted. After calculations were completed, the postprocess stage was automatically initiated. First, the dose and LET_d_ files created from each parallel simulation or batch, typically 50 files per field, were read into dose and LET_d_ grids. LET_d_ and average dose of a voxel were calculated by Eqs. (1) and (2) respectively. Dose_i_ and LET_i_ values represent dose and LET_d_ values of the *i*th batch.

(1) $$\bar{LET_{d}}=\frac{\sum_{i=1}^{N}LET_{i}xDose_{i}}{\sum_{i=1}^{N}Dose_{i}}$$

(2) $$\bar{D}=\frac{\sum_{i=1}^{N}Dose_{i}}{N}$$

The conversion of Monte Carlo scored dose, usually tallied as either energy per voxel or energy per voxel per particle, requires a correction factor that scales the scored dose by the number of particles simulated and MU in the plan file.^23^ For the calculation of absolute dose, a relationship was derived between dose per MU in a single spot measurement and dose per particle for a single spot simulation. A PTW 34070 Bragg Peak Chamber was used to measure dose at a depth of 2 cm in water for 96 nominal beam energies. Doses per MU value were calculated from measurements. The experimental setup was modeled using TOPAS, with dose scoring occurring in the detector volume and being normalized by the number of particles simulated. The ratio of the two values was the number of particles per MU at each of the 96 clinical energies used at our center. The ratio was used in the dose conversion and assignment of particles per energy in the simulation.

The final step of postprocessing was to write the dose and LET_d_ files into the DICOM format. The new DICOM files could be imported into a medical imaging software or TPS to compare them with the TPS‐calculated dose or, in the case of LET, on its own.

### 2.E Integration of software

Because in a generalized scenario the independent calculations will require at least two different applications or software packages (i.e., the TPS and the independent calculation), there needs to be in place a method to get each piece of the automation process to communicate with the previous and subsequent steps. We developed a set of ‘listener' scripts in Perl that would monitor each phase of the automation and appropriately queue the next step after the successful completion of the prior step. When the DICOM export occurs within the TPS and the automation is initiated with the execution of the ESAPI script, the presence of files on our virtual server indicates to the Perl scripts that a calculation is ready to begin. The Perl scripts execute form the command line each MATLAB script used in preprocessing, initiate the bash script used for dispatching the TOPAS calculation, then execute the MATLAB scripts used for postprocessing. At each step text is written to a log file for tracking of jobs. The Perl script is set to check the status of job IDs on the server every 10 min. When the entire calculation is finished and the Perl script sees that the job IDs associated with the calculation are completed, an e‐mail notification is sent to the user that dispatched the jobs.

### 2.F Validation

The accuracy of the system and integrity of the data exchange in the automation was validated against patient‐specific QA measurements. Validation of the beamline model (i.e., comparison of ranges, spot width, etc.) was previously performed and reported on Ref. [24, 25]. Briefly, the system validation and patient‐specific QA comparison performed in this work entails measurements conducted for at least two depths per patient, per treatment field. The setup consists of a MatriXX PT (IBA Dosimetry, Bartlett, TN, USA) ionization array positioned at radiation isocenter. Water equivalent slabs are placed on top of the array to measure dose at depth. Validation was performed for each of our three unique beam models: gantry room model, fixed beam model, and mini‐beam model. In addition to each of the three models, measurement based comparisons were performed for cases with and without the use of our nozzle mounted range shifter. For cases involving a nozzle mounted range shifter, the underlying model remained unchanged, and 40 mm ABS resin slab was placed in the simulated nozzle for explicit transport. Testing of all beam models and scenarios with and without a range shifter present ensured the accuracy of the models and data processing for the commissioned clinical treatment strategies used at our clinic.

The measured planar dose distribution was extracted from the MatriXX PT software and compared to the calculated, 3D Monte Carlo dose distribution by using an in‐house developed 3D gamma comparison^26^ code. The criteria was a global 3%/3 mm with a goal of greater than 95% of tested pixels passing.

## 3 RESULTS AND APPLICATIONS

The developed automated MC system can be used for verifying the treatment plan dose as well as for dose‐weighted LET calculations. Exact reductions in human‐hours are difficult to quantify due to the dependence on user familiarity with the preautomation workflow. Before implementation, a familiar user spent 1–2 hr on necessary data preparation and processing. For the unfamiliar user, the process can be prohibitively complex and as a result unusable. After implementation, user interaction was reduced to 2–3 min, all of which were spent within the TPS.

In the following sections, we present validation results and illustrate two patient cases to demonstrate the two applications—dose verification and LET calculations—of the automated system.

### 3.A Validation

A total of 160 comparisons between measurement and Monte‐Carlo calculated doses were performed at depths of 1cm through 15 cm. Of the 160 comparisons, 71 were performed for the standard gantry model with no beam accessories in place and 24 were performed with the addition of the nozzle mounted range shifter in place, 20 were performed for the fixed beamline, and 45 were performed for fixed mini‐beamline. A complete breakdown of passing rates for each validated scenario is included in Table 1. Among the 160 comparisons, the average gamma passing rate was 99.4%. The passing rates ranged from 96.3% to 100%. All fields met the threshold of 95% of pixels passing the 3%/3mm global criteria.

**Table 1:** The average 3D gamma passing rates for a ± 3%/3 mm criteria are reported for each measurement scenario. The gantry, fixed, and fixed mini‐beamlines are distinct beamline models. The nozzle mounted range shifter was explicitly modelled in the TOPAS simulations.

Model	Gamma passing rate (Average/Minimum)
Gantry Beamline	99.2%/96.3%
Gantry Beamline w/ Range Shifter	99.3%/96.5%
Fixed Beamline	99.5%/98.2%
Fixed Mini‐beamline	99.8%/97.1%

### 3.B Dose comparison

**Fig. 3:**
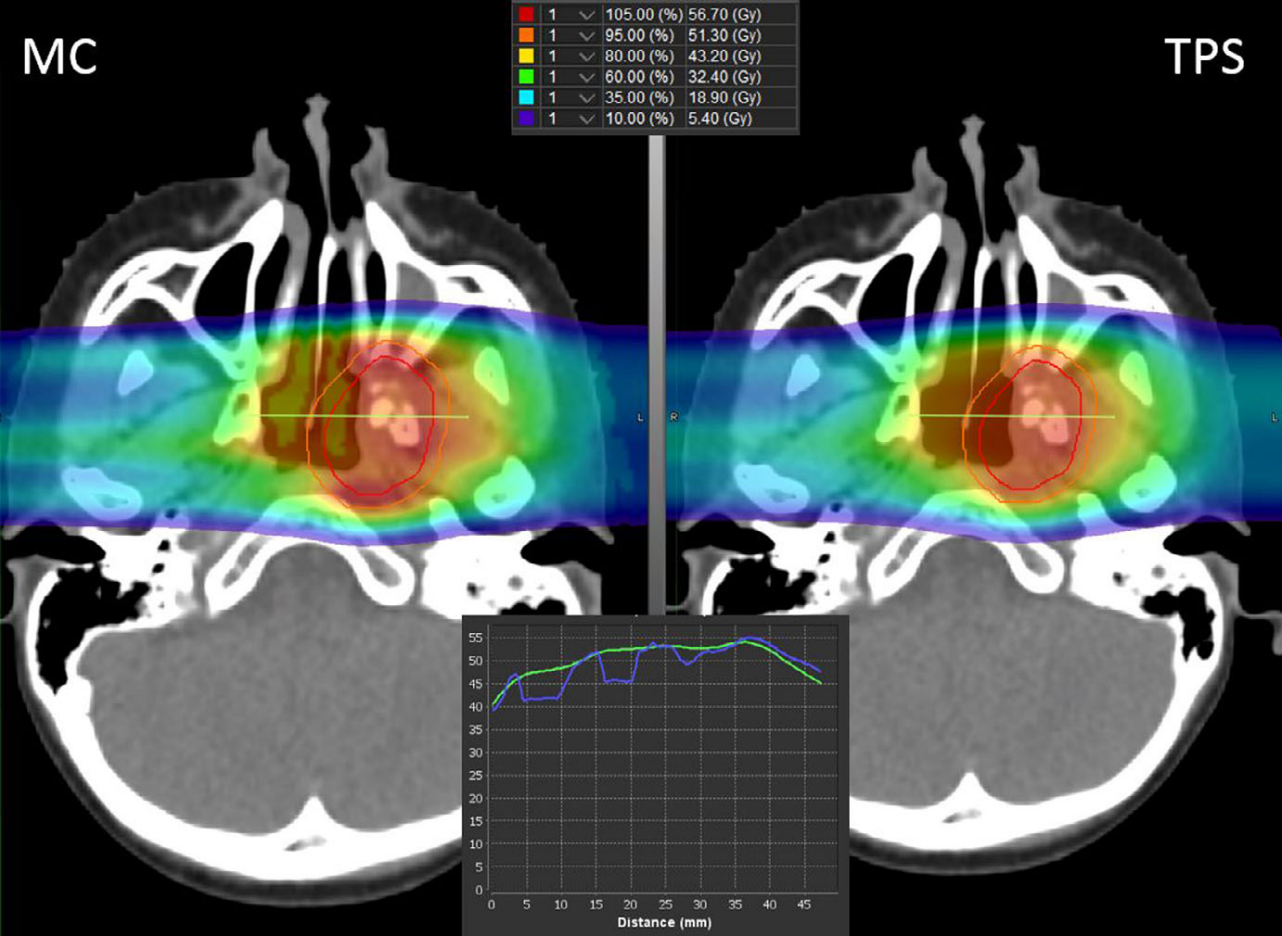
Dose Comparison of treatment planning systems (TPS) and MC – Axial slice of a patient's CT, with MC (left) and TPS (right) dose shown as a color wash. The red and orange contours represent gross tumor volume and clinical target volume respectively. An inset of a dose profile through the target volumes shows the TPS dose (green) and MC dose (blue)

A pediatric patient with Ewing sarcoma of the infratemporal fossa treated with two lateral beams was chosen to calculate the MC dose. This tumor site was selected because of presence of heterogeneities near the target. The MC dose was calculated using the automated MC system and compared with the TPS dose. Figure 3 shows an axial slice of the patient's CT with an overlay of MC and TPS dose calculations.

Figure 3 shows that the target site is closer to the ethmoid sinus, and the bone‐air interface introduces large heterogeneities along the path of the proton beam. Heterogeneities along the beam path make it difficult for the analytical algorithm used in the TPS to include accurate multiple coulomb scattering effects when calculating dose distributions. There is an observable reduction in the predicted dose by the MC system in the sinus and at the interface of the tissue. Thus, using the automated MC system as a second check can help evaluate the accuracy of dose calculations, especially around heterogeneities.

### 3.C LET_d_ calculations

Although LET distributions in proton radiation therapy have been extensively studied,^13^, ^14^ no study has quantitatively correlated LET values, volume effects, or spatial distribution with the likelihood of patients developing radiation necrosis or other radiation‐induced toxicities. In the absence of quantitative guidelines on LET distributions, we propose using a qualitative approach to minimize LET‐related biological uncertainty.

**Fig. 4:**
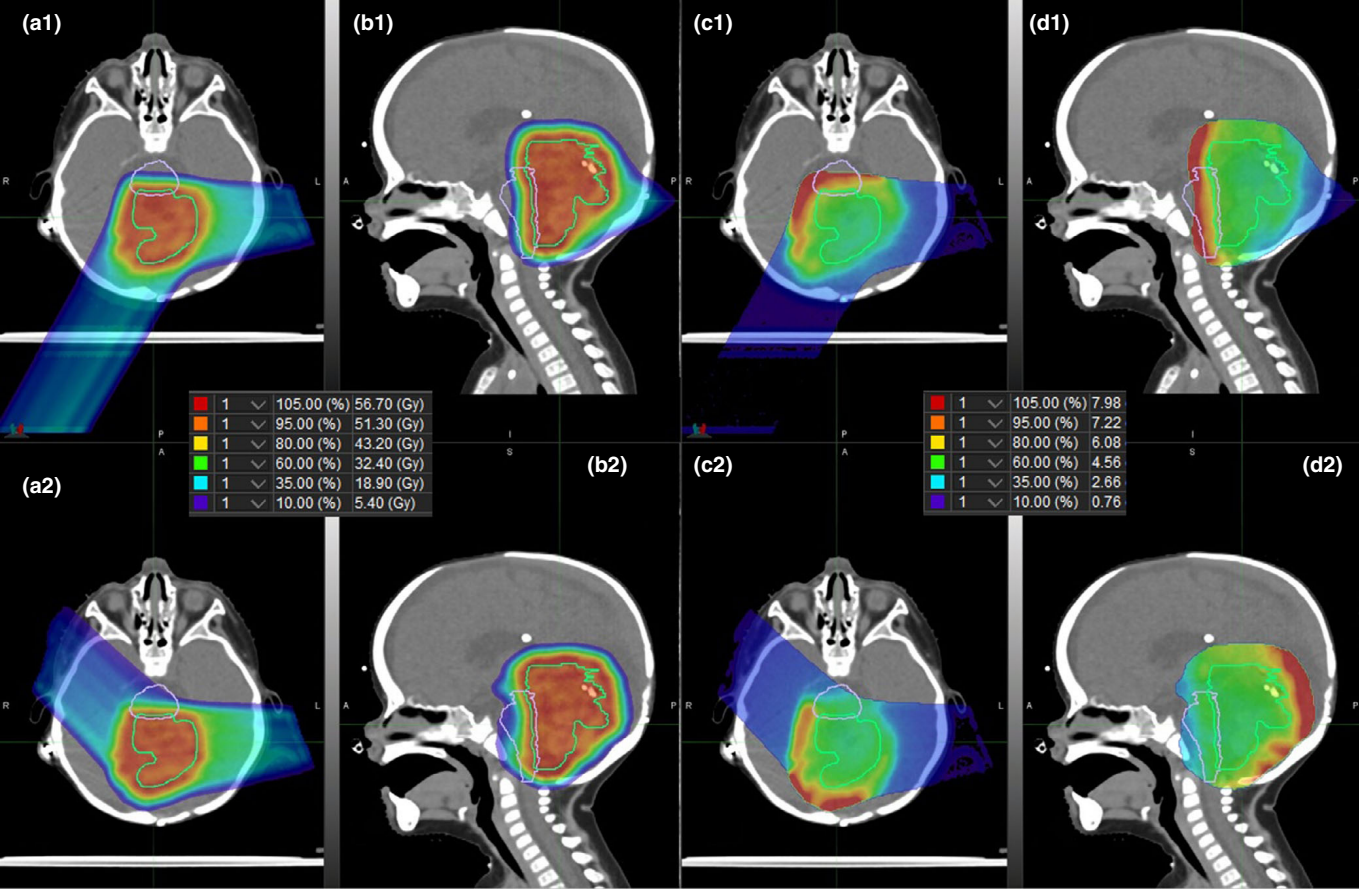
Linear energy transfer (LET) Comparison of Two Plans – Dose and LET_d_ distributions in transverse (a and c) and sagittal (b and d) planes for two proton treatment plans for a pediatric patient with atypical teratoid rhabdoid tumor. Dose distributions are shown in (a) and (b) with inset legend, and LETd distributions are shown in (c) and (d) with inset legend in units of keV/micron. The LETd was masked to in‐field, defined as greater than 10% of prescription dose, only. Plan 1 was optimized with lateral and posterior oblique beams, and plan 2 was optimized with lateral and anterior oblique beams. The green contour is the clinical target volume and the purple contour is the brainstem

Figure 4 shows sagittal images of a pediatric patient with an atypical teratoid rhabdoid tumor (ATRT) treated with proton therapy. Images are color wash maps of dose (a and b) and LET_d_ (c and d) distributions for two unique treatment plans (1 and 2) designed to deliver the same physical dose and meet the same clinical constraints. The only change in plan was the beam angles used to cover the target. In the images 1c and 1d, areas of increased LET_d_ are present in the posterior part of the brainstem. After the beam orientation is changed, images 2c and 2d show a reduction in LET_d_ in the brainstem. This reduction in LET_d_ was achieved without compromising the physical dose distribution traditionally used for the plan evaluation.

We envision a workflow in which treatment plans can be prospectively assessed for biological robustness (i.e., how uncertainties related to LET_d_ maps may affect plan quality) during the treatment planning process. Individual plans can be evaluated, or suitable plans can be compared to evaluate differences in LET distributions. In our early use of the tool for these types of comparisons we have focused on identifying areas of overlapping high dose and high LET_d_, defined by us as dose greater than 80% of the prescription dose and LET_d_ greater than 6 keV/micron. When the overlapping these two overlapping areas is in an area of high functional importance (e.g., the brainstem), we will choose beam angles or manipulate spot placement to mitigate end of range effects while preserving dosimetric quality of the plan. As we continue to collect data through the use of the automated Monte Carlo tool, we plan to build a repository of LET_d_ distributions for use in retrospective studies that can improve our understanding of biological risks associated with LET_d_.

## 4 DISCUSSION

We developed an MC‐based secondary check system that can independently verify treatment plan doses while also calculating LET_d_ to evaluate biological robustness and research data collection. Critical to deploying such a tool in a clinical environment are ease of use, minimal impact on workflow, and reduced chance for human error. To this end, we developed an automated framework that can be initiated by physicists or dosimetrists within the TPS.

For ease of use, it is important that clinical users be able to deploy the tool without having detailed knowledge of command line deployment, function dependencies, and integration with institutional resources such as the HPC facility. By using the Eclipse Scripting API, calculations can be initiated with just a few mouse clicks in an entirely graphical manner. Otherwise, it would be unrealistic to expect clinical team members to routinely use the tool.

It is important to note that TOPAS is not a fast Monte‐Carlo code. We felt the tradeoff in performance was more than made up for in having a free, publicly available, well benchmarked, Monte Carlo code. In our implementation a single patient calculation can be performed in about 16 hr (compared to minutes for the analytical calculation within the TPS). When calculating a treatment plan we queue it up at the time the patient's plan is ready for QA. This ensures that when the QA is reviewed the next day, a secondary calculation will also be available for review. What is feasible for a clinic will be highly dependent on hardware resources available as well as personnel resources should in‐house Monte Carlo methods be chosen over TOPAS. We felt that our choices made the example of workflow most broadly applicable.

We demonstrated two clinical cases in which the automated MC system can be helpful. The first case was that of a pediatric patient with Ewing sarcoma of the infratemporal fossa treated with two lateral beams. The MC‐ and TPS‐calculated dose distributions were compared. In this extreme example of traversing tissue heterogeneities, there was disagreement between the two dose calculations at tissue boundaries. Understanding where the two dose calculations differ may not be so intuitive in other cases, and therefore a 3D prediction of dose from an MC calculation is especially valuable to assess the quality of the treatment plan.

The dosimetric comparison between TPS and MC calculations did not address actionable levels at which intervention is recommended. We feel this is best handled on a case‐by‐case basis through a discussion between the dosimetrist, physicist, and physician involved in the plan. When the discrepancy is due to heterogeneous tissues, the ability to correct for TPS calculation accuracy is limited as that is driving the optimization of spot placement and weighting during the planning process. In these scenarios we typically consider alternate beam paths that might limit the extent to which heterogeneous tissue is traversed. Alternatively, the renormalization of the dose can be used to ensure minimum coverage or maximum hot spot constraints are met.

The second case (Fig. 4) was that of a pediatric patient with ATRT. The choice of beam delivery angle was evaluated based on resultant LET_d_ maps, without compromising dose distribution. Even though this result was not clinically used, it is a good example to demonstrate that critical organs or sites could be spared by taking LET_d_ distributions into account during treatment planning. The concept of biologically robust planning by using different optimization strategies or treatment angles has been previously explored.^15^ Our example demonstrates how biological robustness can be evaluated within the clinical workflow.

## 5 CONCLUSION

The main purpose of developing the automated MC system was to reduce the time spent on manual operations pre‐ and postsimulation and increase access to an independent MC system by removing barriers to use, most importantly detailed knowledge of how to deploy the calculations in a stepwise manner. By eliminating most of the human interaction required to run a calculation, we reduced the potential for human error in data manipulation, processing, and deployment while increasing access to an important tool for quality control of treatment plans. Although our method does not reduce the actual calculation time, it removes human factor‐related barriers to wide‐scale implementation in the clinic. Our approach toward automating secondary Monte‐Carlo calculations can be adapted to other treatment planning systems or Monte‐Carlo codes by taking advantage of the increased access to TPS coding through vendor provided APIs. The approach stands as a valuable demonstration of a framework towards bringing in tools that were previously relegated for research only and using them as a valuable source of information in a quality control program.

## CONFLICT OF INTERESTS

The authors have no conflicts of interest to report.
